# Malocclusion and its relationship with oral health-related quality of life in patients with eating disorders

**DOI:** 10.1590/2177-6709.27.2.e2220305.oar

**Published:** 2022-06-10

**Authors:** Fernando Yamamoto CHIBA, Erika Kiyoko CHIBA, Suzely Adas Saliba MOIMAZ, Doris Hissako MATSUSHITA, Artênio José Ísper GARBIN, Cléa Adas Saliba GARBIN

**Affiliations:** 1 Universidade Estadual de São Paulo (UNESP), Faculdade de Odontologia, Departamento de Odontologia Preventiva e Restauradora (Araçatuba/SP, Brazil).

**Keywords:** Malocclusion, Anorexia nervosa, Bulimia nervosa, Quality of life

## Abstract

**Objective:**

To evaluate the prevalence and severity of malocclusion and its impact on oral health-related quality of life (OHRQoL) and self-reported satisfaction of patients with anorexia and bulimia nervosa.

**Methods:**

The sample consisted of sixty women who attended a specialized mental health clinic of a Brazilian medical school. Participants were distributed into two groups: patients with anorexia and bulimia nervosa (ABN; n=30) and control patients without eating disorders (CN; n=30). The dental occlusion was evaluated by the Dental Aesthetic Index; the OHRQoL was assessed using the OHIP-14 questionnaire; and the self-reported satisfaction with the appearance of teeth, speech ability and chewing was obtained by interviews.

**Results:**

Severe and very severe malocclusion were observed in 26.67% and 46.67% of patients in the ABN group, respectively, while the CN group showed 80.00% of patients without abnormality/mild malocclusion. ABN group showed a higher proportion of patients (*p* < 0.05) with tooth loss, spacing in the region of incisors, maxillary misalignment, and mandibular misalignment in relation to CN group. ABN group presented lower (*p*< 0.05) OHRQoL and self-reported satisfaction with the appearance of teeth, speech ability and chewing, compared to the CN group. There was a significant positive correlation (*p*< 0.05) between the Dental Aesthetic Index and OHIP-14 scores in the ABN group.

**Conclusions:**

The prevalence of severe malocclusion in ABN group was high, with a negative impact on OHRQoL and self-reported satisfaction with the chewing ability, speech ability and appearance of teeth.

## RESUMO

**Objetivo:**

Avaliar a prevalência e gravidade da má oclusão e seu impacto na qualidade de vida relacionada à saúde bucal (QVRSB) e a satisfação autorreferida de pacientes com anorexia e bulimia nervosa.

**Métodos:**

A amostra foi composta por 60 mulheres que compareceram a uma clínica especializada em saúde mental de uma faculdade brasileira de Medicina. Os participantes foram distribuídos em dois grupos: pacientes com anorexia e bulimia nervosa (ABN; n=30) e pacientes sem distúrbios alimentares (CN; n=30). A oclusão dentária foi avaliada pelo Índice de Estética Dental; a QVRSB foi avaliada usando o questionário OHIP-14; e a satisfação autorreferida com a aparência dos dentes, capacidade de fala e mastigação foi avaliada por meio de entrevistas.

**Resultados:**

Má oclusão grave e muito grave foi observada em 26,67% e 46,67% dos pacientes no grupo ABN, respectivamente, enquanto o grupo CN apresentou 80,00% dos pacientes sem anormalidade/má oclusão leve. O grupo ABN apresentou maior proporção de pacientes (*p*<0,05) com perda dentária, espaçamento na região dos incisivos, desalinhamento maxilar e desalinhamento mandibular, em relação ao grupo CN. O grupo ABN apresentou menor (*p*<0,05) QVRSB e satisfação autorreferida com a aparência dos dentes, capacidade de fala e mastigação, em comparação ao grupo CN. Houve uma correlação positiva (*p*<0,05) entre o Índice de Estética Dental e o escore do OHIP-14 no grupo ABN.

**Conclusões:**

A prevalência de má oclusão grave no grupo ABN foi alta, com impacto negativo na QVRSB e satisfação autorreferida com a aparência dos dentes, capacidade de fala e mastigação.

## INTRODUCTION

Anorexia and bulimia nervosa are eating disorders characterized by fear of gaining weight, extreme eating behaviors aimed at reducing weight and marked distortion of body self-image.^1^ These conditions may be associated with alterations in mental, systemic and oral health, and the understanding of its effects, as well as the control and treatment of these complications are of fundamental importance, since they may present relevant repercussions on the quality of life and in the treatment of eating disorders.^2^

Eating disorders are characterized by changes in eating behavior that may be associated with damage to dental tissues and impairment in oral health.^3^ The most commonly observed oral changes are dental erosion, tooth sensitivity, dental caries, decreased salivary flow and gingival bleeding.^4^ ^-^ ^7^ These changes may be related to an imbalance in dental occlusion, and may contribute to the development of malocclusion and impairment of dental aesthetics, functional performance, as well as systemic health.^8^ Orthodontic treatment aims to reestablish oral function and health as a final objective, however, the psychological and social effects of orthodontic interventions represent important reasons for seeking treatment.^9^

In this context, it is important to consider the impact of malocclusions on quality of life, since these conditions may be related to impairment of speech and chewing functions, impair the psychological and social condition of individuals and influence the degree of satisfaction with oral condition.^10^ In addition, it is known that individuals with anorexia and bulimia nervosa present poorer quality of life, compared to individuals without eating disorders.^11^

Considering that oral health is an important component that interferes with quality of life and the scarcity of studies about malocclusions in individuals with eating disorders, this study aimed to evaluate the prevalence and severity of malocclusion and its impact in the oral health-related quality of life (OHRQoL) and the self-reported satisfaction of patients with anorexia and bulimia nervosa.

## MATERIAL AND METHODS

This quantitative cross-sectional study was performed on patients attended in a specialized mental health clinic of a Brazilian medical school, in 2018. The sample consisted of sixty women distributed into two groups: control patients without eating disorders (CN group; n=30) and patients with medical history of anorexia and bulimia nervosa diagnosed by psychiatrists of a specialized mental health clinic (ABN group; n=30).

The ABN group included individuals who met the following eligibility criteria: patients with current diagnosis of eating disorders and patients who did not abandon the psychiatric treatment. Exclusion criteria were applied to both groups as follows: patients with a history of accidents that resulted in tooth loss and patients who were currently undergoing orthodontic treatment.

The sample size was determined based on data from a pilot study, considering the estimated proportion of malocclusion as the primary outcome. It was used a Z-test applied for two proportions, with a reliability of 95%, a margin of error of 5%, and a test power of 80%, resulting in a minimum sample size of 27 participants per group. All patients with eating disorders undergoing treatment at the Specialized Mental Health Clinic were invited to participate in the research, totaling 30 patients in each group. Participants in the CN group were selected from the companions of patients in the ABN group following a psychiatric consultation. This was done to maintain sample homogeneity and avoid bias, due to significant differences in socioeconomic characteristics between groups. The patients who agreed to participate in the study were submitted to oral clinical examination, evaluation of the oral health-related quality of life and assessment of self-reported satisfaction with the appearance of teeth, speech ability and chewing.

### ETHICAL ASPECTS

The research was approved by the Human Research Ethics Committee (CAAE: 80497417.1.0000.5420) of *Universidade Estadual de São Paulo* and carried out in accordance with the Helsinki Declaration. Informed consent was obtained from all individual participants included in the study.

### ORAL CLINICAL EXAMINATION

The Dental Aesthetic Index (DAI) was used to evaluate the prevalence of malocclusions, its severity and the need for treatment.^12^ The examinations were carried out in the specialized mental health clinic of the medical school, in isolated rooms with good lighting conditions, using a chair, a mouth mirror and a WHO periodontal probe. The data were collected by a blind previously calibrated single dental surgeon. The calibration process consisted of a theoretical study involving the study of the DAI index and the performance of clinical examinations. The Kappa test for occlusal condition assessment was performed to verify intra-examiner agreement, and a value of 0.90 was obtained.

### EVALUATION OF ORAL HEALTH-RELATED QUALITY OF LIFE (OHRQOL)

OHRQoL was evaluated by the OHIP-14 questionnaire.^13^ The questionnaire contains 14 questions and evaluates seven dimensions of the impact of oral conditions on individual’s quality of life, including functional limitation, physical pain, psychological discomfort, physical disability, psychological disability, social disability, and handicap. The response format follow a Likert-type frequency scale, as described: never = 0, hardly ever = 1, occasionally = 2, fairly often = 3, and very often = 4. The additive method was used to calculate the score of each dimension. Thus, the OHIP-14 score for each of the seven dimensions ranged from 0 to 8, with higher scores indicating a poorer OHRQoL.

### ASSESSMENT OF SELF-REPORTED SATISFACTION

A structured questionnaire was used to assess patients’ satisfaction with the appearance of teeth, speech ability and chewing. Answers were classified into two categories: satisfied (satisfied and very satisfied) and dissatisfied (dissatisfied and very dissatisfied).

### STATISTICAL ANALYSES

The data about the occlusal condition, orthodontic treatment need, OHIP-14 score, and self-reported satisfaction were described by using descriptive statistics. The values of age, Dental Aesthetic Index score and OHIP-14 score for each dimension were analyzed for normality using the Shapiro-Wilk test, and the Mann-Whitney test was used to compare the groups. The correlation between Dental Aesthetic Index score and total OHIP-14 score was analyzed by the Spearman correlation test. Two-proportion test was used to compare the proportion of patients with very severe or disabling malocclusion, and patients with occlusal alterations according to the Dental Aesthetic Index criteria. The chi-square test was performed to evaluate the association between eating disorder and the degree of satisfaction with speech ability, chewing ability and aesthetic appearance of teeth. The results were expressed as the mean ± standard deviation, and differences between groups were considered significant at *p* < 0.05. Data analysis was performed using GraphPad Prism 7.0 software.

## RESULTS

There was no difference in the age between groups (CN group = 28.93 ? 9.77; ABN group = 31.13 ? 12.72). Table 1 shows that almost half of patients in the ABN group had very severe or disabling malocclusion, while in the CN group, most patients showed without abnormality or with mild malocclusion. The majority of patients of the ABN group presented orthodontic treatment need classified as highly desirable or indispensable. The proportion of patients with very severe or disabling malocclusion was significantly higher (*p* < 0.0048) in the ABN group than in the CN group. 

**Table 1:** Occlusal condition and orthodontic treatment need in CN and ABN groups, according to the Dental Aesthetic Index (DAI).

DAI score	Occlusal condition	Treatment need	CN group		ABN group	
			n	%	n	%
≤ 25	Without abnormality or mild malocclusion	Little or no need	24	80.00	2	6.67
26 to 30	Defined malocclusion	Elective	2	6.67	6	20.00
31 to 35	Severe malocclusion	Highly desirable	0	0.00	8	26.67
≥ 35	Very severe or disabling malocclusion	Indispensable	4	13.33	14	46.67
Total			30	100.00	30	100.00

The proportion of patients with alterations in the Dental Aesthetic Index (DAI) criteria is demonstrated in the Table 2. ABN group showed a higher proportion of patients (*p*< 0.05) with upper teeth loss, lower teeth loss, spacing in the region of incisors, anterior maxillary misalignment, and anterior mandibular misalignment in relation to CN group. There was no difference in the proportion of patients with alterations in crowding in the incisor region, diastema, anterior maxillary overjet, anterior mandibular overjet, anterior open bite and molar relationship.

**Table 2:** Distribution of patients according to the presence of alterations in the Dental Aesthetic Index (DAI) criteria in the CN and ABN groups.

DAI components	CN group				ABN group				p-value
	Presence		Absence		Presence		Absence		
	n	%	n	%	n	%	n	%	
DENTITION									
Upper teeth loss	2	6.67	28	93.33	8	26.67	22	73.33	0.0377
Lower teeth loss	2	6.67	28	93.33	8	26.67	22	73.33	0.0377
SPACE									
Crowding	16	53.33	14	46.67	18	60.00	12	40.00	0.6023
Spacing	2	6.67	28	93.33	10	33.33	20	66.67	0.0098
Diastema	2	6.67	28	93.33	2	6.67	28	93.33	1.0000
Maxillary misalignment	8	26.67	22	73.33	26	86.67	4	13.33	< 0.0001
Mandibular misalignment	8	26.67	22	73.33	28	93.33	2	6.67	< 0.0001
OCCLUSION									
Anterior maxillary overjet	2	6.67	28	33.33	3	10.00	27	90.00	0.6404
Anterior mandibular overjet	0	0.00	30	100.00	2	6.67	28	93.33	0.1503
Anterior open bite	0	0.00	30	100.00	0	0.00	30	100.00	1.0000
Molar relationship	4	13.33	26	86.67	2	6.67	28	93.33	0.3894

Two-proportion test was used to compare the groups.

It was observed that the ABN group showed a significantly higher (*p*<0.05) Dental Aesthetic Index score and total OHIP-14 score compared to the CN group (Table 3). There was a significant positive correlation (r=0.8461; *p*<0.0001) between the DAI score and the OHIP-14 score in the ABN group.

**Table 3:** Dental Aesthetic Index (DAI) and total OHIP-14 scores in CN and ABN groups.

Variables	CN group	ABN group	p-value
DAI score	21.33 ± 8.58	38.33 ± 10.65	<0.0001
OHIP-14 score	3.67 ± 4.44	22.2 ± 15.71	<0.0001

Values are presented as mean ± standard deviation. Mann-Whitney test was used to compare the groups.

The ABN group presented significantly higher scores (*p*<0.05) in the dimensions of functional limitation, physical pain, psychological discomfort, physical disability, psychological disability, social disability, and handicap, compared to the CN group (Table 4).

**Table 4:** Scores of OHIP-14 dimensions in CN and ABN groups.

OHIP-14 dimensions	CN group	ABN group	p-value
Functional limitation	0.07 ? 0.25	2.00 ± 2.20	< 0.0001
Physical pain	0.87 ± 0.97	3.07 ± 2.36	0.0003
Psychological discomfort	1.53 ± 1.48	4.87 ± 2.73	< 0.0001
Physical disability	0.13 ± 0.35	2.53 ± 2.54	< 0.0001
Psychological disability	0.73 ± 1.31	3.40 ± 2.77	< 0.0001
Social disability	0.33 ± 1.27	1.93 ± 2.45	0.0008
Handicap	0.07 ± 0.25	1.80 ± 2.11	< 0.0001

Values are presented as mean ± standard deviation. Mann-Whitney test was used to compare the groups.

It was observed that the degree of satisfaction with the oral condition was worse in the ABN group when compared to the CN group. There was a significant association between the presence of eating disorders and dissatisfaction with speech ability (*p*<0.0001), chewing ability (*p*<0.0001) and aesthetic appearance of teeth (*p*<0.0001) (Table 5).

**Table 5:** Self-reported satisfaction with speech ability, chewing ability and appearance of teeth in CN and ABN groups.

Group	Self-reported satisfaction	Speech ability		Chewing ability		Appearance of teeth	
		n	%	n	%	n	%
CN	Satisfied	30	100.00	24	80.00	28	93.33
	Dissatisfied	0	0.00	6	20.00	2	6.67
	Total	30	100.00	30	100.00	30	100.00
ABN	Satisfied	16	53.33	6	20.00	4	13.33
	Dissatisfied	14	46.67	24	80.00	26	86.67
	Total	30	100.00	30	100.00	30	100.00

## DISCUSSION

In this research, it was observed that the ABN group presented a worse occlusal condition evaluated using the Dental Aesthetic Index, and poorer oral health-related quality of life, as assessed using the OHIP-14 questionnaire. These changes were accompanied by decreased self-reported satisfaction with speech ability, chewing ability and aesthetic appearance of teeth.

It was observed that data related to age were similar between the two groups. Thus, it is possible to suggest that the differences in the occlusal condition, oral health-related quality of life, and self-reported satisfaction observed in the present study were not caused by this variable.

Eating disorders can negatively affect the oral condition of patients and are associated with the development of dental caries, dental erosion, hyposalivation, and gingivitis.^3^ ^,^ ^14^ Excessive consumption of carbonated drinks as appetite suppressants, gastric reflux, frequent self-induced vomiting and nutritional deficiency may contribute to the development of these changes.^15^ It should be noted that there are few reports in the literature about malocclusion in patients with anorexia and bulimia nervosa. A study carried out in patients with anorexia and bulimia nervosa hypothesized that insertion and finger pressure used to induce frequent vomiting could result in tooth movement and development of malocclusion.^16^ However, the presence of orthodontic abnormality was not associated with reports of self-induced vomiting, which suggested that digital pressure was not the causal factor. 

The progression of dental caries and periodontal disease can lead to tooth loss and is associated with malocclusion.^17^ ^,^ ^18^ Indeed, in the present study, it was observed that a great proportion of patients in the ABN group showed tooth loss of incisors, canines and/or permanent premolars in the upper and lower dental arches. Tooth loss may result in the alteration of tooth positions relative to the basal bone of the alveolar process and adjacent teeth.^19^ Thus, it is possible to suggest that tooth loss may have contributed to the worsening of occlusal condition related to alterations in spacing, maxillary and mandibular misalignment.

It was observed that the patients in the ABN group showed impairment in OHRQoL and that the severity of the malocclusion is correlated with the worsening in the quality of life of these individuals. These findings are in agreement with a study that verified the negative impact of malocclusion on OHRQoL in adults and the improvement of this variable through orthodontic treatment.^20^ The present results are in according to a recent systematic review that verified that the quality of life of patients with anorexia and bulimia nervosa was significantly worse in comparison with healthy populations.^11^ In addition, these conditions are associated with a high rate of hospitalization, ambulatory care and emergency consultations, and increased health costs, highlighting the need to improve the understanding about the factors influencing the quality of life of these individuals.^11^

It should be noted that the ABN group presented the worst result in all dimensions evaluated by the OHIP-14, which include functional limitation, physical pain, psychological discomfort, physical disability, psychological disability, social disability, and handicap. Thus, it is possible to suggest that the oral condition of these patients negatively influences the performance of several activities, such as daily tasks, development of interpersonal relations, and eating behavior, resulting in worsening of life quality. This finding is very relevant because it is known that eating disorders are often associated with the comorbidities of depression and anxiety disorders, and that individuals with eating disorders have substantial difficulties in recognizing and regulating emotions.^21^ ^,^ ^22^ Moreover, it is suggested that decrease in quality of life of these patients may have an important effect on the severity of psychological symptoms and risk of suicide.^22^ Therefore, the control of factors that may influence the symptoms of depression and anxiety can play a prominent role in restoring the mental health of these patients.^23^

There is a complex relationship between oral health maintenance and eating disorders. Studies suggest that there are patients with eating disorders who may be less interested in maintaining oral health due to their depressive condition, while there are patients who may present a compulsive oral hygiene practice.^24^ ^,^ ^25^ The negative effect of malocclusion and its impact on quality of life were perceived in the ABN group self-reported satisfaction analysis, in which approximately half of the patients in this group had dissatisfaction with speech ability, while more than three quarters showed dissatisfaction with chewing ability and aesthetic appearance of teeth.

The present results are in agreement with studies that verified that malocclusion is associated with reduced speech capacity and lower efficiency of chewing ability, highlighting that orthodontic deviations should be considered in oral health planning, to identify risk groups and to improve health services.^26^ ^,^ ^27^ The relationship between malocclusion and the lower degree of satisfaction with the aesthetic appearance of teeth has been reported in previous studies, considering that orthodontic treatment may improve body image and, especially, facial image.^26^ ^,^ ^28^

Restoration of weight and nutritional status are key elements in the treatment of eating disorders.^29^ In this context, the data about self-reported satisfaction with chewing ability, functional limitation, physical pain and physical disability revealed a worrying situation. It was observed that patients of the ABN group presented unsatisfactory diet, need to interrupt meals, chewing discomfort and worsening in sense of taste due to oral problems. Systematic review has shown that malocclusions cause a decrease in masticatory performance, especially in relation to the reduction of occlusal contact area.^30^ Thus, it is possible to suggest that the occlusal condition may have an important negative impact on the nutritional rehabilitation of patients with anorexia and bulimia nervosa.

In this research, all patients in the ABN group who agreed to participate in the study were female. Thus, to maintain the homogeneity of the sample, men were also not included in the CN group. However, even though the sample is composed only of women, this research presents significant results because the literature demonstrates that eating disorders have greater prevalence in women than in men^2^ ^,^ ^4^. In a cross-sectional study, the outcome and the exposure factor are evaluated simultaneously, so that it is not possible to evaluate possible local etiological effects acting over a period of time, which can be considered a limitation of the present study. 

## CONCLUSIONS

The prevalence of severe malocclusion in ABN group was high, with a negative impact on OHRQoL and self-reported satisfaction with the chewing ability, speech ability and appearance of teeth. The results of this study reinforce the importance of the participation of the dentist in an interdisciplinary approach for treatment of patients with anorexia and bulimia nervosa.
